# Light Inhibition of Shoot Regeneration Is Regulated by Endogenous Abscisic Acid Level in Calli Derived from Immature Barley Embryos

**DOI:** 10.1371/journal.pone.0145242

**Published:** 2015-12-15

**Authors:** Kazuhide Rikiishi, Takakazu Matsuura, Yoko Ikeda, Masahiko Maekawa

**Affiliations:** Institute of Plant Science and Resources, Okayama University, Kurashiki, Okayama, Japan; Estación Experimental del Zaidín (CSIC), SPAIN

## Abstract

Shoot regeneration in calli derived from immature barley embryos is regulated by light conditions during the callus-induction period. Barley cultivars Kanto Nijo-5 (KN5) and K-3 (K3) showed lower efficiency of shoot regeneration in a 16-h photoperiod during callus-induction than those in continuous darkness, whereas shoot regeneration was enhanced in cultures under a 16-h photoperiod in Golden Promise (GP) and Lenins (LN). These cultivars were classified as photo-inhibition type (KN5 and K3) or photo-induction type (GP and LN) according to their response to light. Contents of endogenous plant hormones were determined in calli cultured under a 16-h photoperiod and continuous darkness. In photo-inhibition type, higher accumulation of abscisic acid (ABA) was detected in calli cultured under a 16-h photoperiod, whereas calli showed lower levels of endogenous ABA in continuous darkness. However, cultivars of photo-induction type showed lower levels of ABA in calli cultured under both light conditions, similarly to photo-inhibition type in continuous darkness. Exogenous ABA inhibited the callus growth and shoot regeneration independent of light conditions in all cultivars. In photo-inhibition type, lower levels of endogenous ABA induced by ABA biosynthesis inhibitor, fluridone, reduced the photo-inhibition of shoot regeneration. Expression of ABA biosynthesis gene, *HvNCED1*, in calli was regulated by the light conditions. Higher expression was observed in calli cultured under a 16-h photoperiod. These results indicate that ABA biosynthesis could be activated through the higher expression of *HvNCED1* in a 16-h photoperiod and that the higher accumulations of ABA inhibit shoot regeneration in the photo-inhibition type cultivars.

## Introduction

Production of transformants is an important technique for the analysis of gene expression and for the development of next-generation breeding methods. Shoot regeneration from calli and cultured cells is not only a crucial step in the production of transformants; it is also a useful tool for investigating the totipotency of plant cells. Plant hormones auxin and cytokinin are generally used for vegetative propagation and for regeneration procedures. Kraepiel *et al*. [1] reported that the ratio of endogenous levels of auxin to cytokinin is fundamentally important for the regulation of tissue culture traits such as callus growth and shoot regeneration in tobacco cultured cells. In addition, the effects of abscisic acid (ABA) on callus growth and shoot regeneration have been reported [2–7]. Actually, ABA regulates several processes related to plant growth, development, and response to environmental stress. Shoot regeneration and embryogenesis from cultured cells are regulated by an intricate regulatory network involving auxin, cytokinin, and ABA.

Genome-wide analysis revealed that several key factors and genes related to hormonal signal transduction pathway are involved in the regulation of shoot regeneration in Arabidopsis [8–11]. In fact, QTLs controlling shoot regeneration were also investigated in rice [12–15], wheat [16], barley [17, 18], tomato [19, 20], cabbage [21], and sunflower [22]. Recently, Motte *et al*. [23] identified *receptor-like kinase 1* (*RPK1*), which was involved in ABA signal transduction pathway, as a QTL for the regulation of shoot regeneration from calli in Arabidopsis. Furthermore, Zhang *et al*. [24] reported that the expressions of abiotic stress-induced miRNAs, which were involved in the regulation of ABA responses, were down-regulated in embryogenic calli of *Larix leptolepis*. These results indicate that ABA serves an important role in the regulation of shoot regeneration. However, the functions of ABA on tissue culture traits remain unknown because its effects differ among plant species and explant sources.

In Arabidopsis, mutants showing hypersensitivity or insensitivity to ABA have been analyzed. The genes involved in ABA signal transduction (*ABIs*, *ERA*, *ABH*) were identified [25–27]. These genes regulate sensitivity to ABA. In addition, ABA biosynthesis genes were identified. The biosynthesis pathway involved *zeaxanthin epoxidase* (*ZEP*), *9-cis-epoxycarotenoid dioxygenase* (*NCED*), *ABA2*, and *aldehyde oxidase* (*AAO*) [28]. NCED acts at a key enzyme at the final step for ABA production. However, CYP707A encoded ABA 8’-hydroxylase was involved in ABA catabolism and inactivated ABA [29, 30]. ABA accumulation is in fact determined by the balance between biosynthesis and catabolism. In plants, the response to ABA is regulated by sensitivity and accumulation.

Transformation of barley has been achieved using biolistics and Agrobacterium-mediate gene transfer [31, 32]. However, some problems remain for application of gene transfer technology to crop improvement. Genotype dependence is a major problem [33]. In barley, only limited genotypes showing high performances for callus growth and shoot regeneration are transformed efficiently. Improvements of tissue culture traits are important cues for application of gene transfer technology. As explants for gene transfer, several tissues have been examined, such as immature embryos [31, 32], immature inflorescences [34], microspores [35], and shoot meristematic cultures [36] in barley. Immature embryo appears to be used as an explant source with high performance of shoot regeneration. Shoot regeneration efficiency in calli derived from immature embryos showed wide variation among cultivars. Genetic analysis shows that multiple genes in barley regulated shoot regeneration [37, 38]. Shoot regeneration efficiency is in fact determined by physiological and environmental factors such as developmental stages of embryo, medium compositions and light conditions [39, 40]. Light conditions during callus induction affect the shoot regeneration efficiency, although no effects are apparent in light conditions during shoot regeneration. Light is an important cue, not only an energy source, affecting the autotrophic growth of plants. It also serves as a signal regulating gene expression. Light signals are accepted by photoreceptors such as phytochromes (red and far-red), cryptochromes and phototropin (blue), and UVR8 (UV-B). Numerous studies have examined light regulation of gene expression through these photoreceptors [41]. However, the regulatory functions of light remain unknown for the photoregulation of shoot regeneration in calli derived from immature barley embryos.

The present study examines endogenous hormone levels in calli derived from immature embryos cultured in different light conditions (16-h photoperiod and continuous darkness) for barley cultivars showing different responses to light conditions. Barley cultivars are classified as either photo-inhibition or photo-induction types. Those types respectively show higher shoot regeneration efficiency in continuous darkness and a 16-h photoperiod. In the photo-inhibition type, ABA is highly accumulated in calli. We investigate the expression of ABA biosynthesis gene and effects of exogenous ABA on shoot regeneration. In addition, the role of ABA for light regulation of shoot regeneration is discussed.

## Materials and Methods

### Plant materials and tissue culture

In a field of the Institute of Plant Science and Resources of Okayama University in 2009 and 2014, four barley cultivars were grown: Kanto Nijo-5 (KN5), K-3 (K3), Golden Promise (GP), and Lenins (LN). Husk-removed immature seeds were sterilized. Immature embryos (1–1.5 mm long) were excised aseptically. Modified MS medium with maltose (30 g l^-1^) was used as a basal medium [40]. The callus-induction medium was supplemented with 9 μM of 2,4-dichlorophenoxyacetic acid (2,4-D). ABA and fluridone were dissolved in ethanol at 10 mM concentration. These solutions were used as stock solutions. The callus-induction medium was supplemented with ABA (1 and 5 μM) or fluridone (0.1 and 0.5 μM). The regeneration medium was supplemented with 6 μM of IAA and 0.2 μM of zeatin. These media were solidified with 0.2% of Gelrite (Merck and Co. Inc.).

For callus induction, 14 immature embryos were placed on 20 ml of the callus-induction medium in a 90 × 15 mm Petri dish with the scutellum in contact with the medium. After four weeks of incubation, calli were transferred to the regeneration medium after removing shoots and roots derived from zygotic embryos. Shoot regeneration was recorded four weeks later. Petri dishes were kept at 25±2°C. Petri dishes were incubated in a 16-h photoperiod (16-h photo) or continuous darkness (Dark) during four weeks of callus induction. As a light source, white fluorescent lamps (FL40S; Toshiba Corp.) were used. The light intensity was 61.5 μmol m^-2^ s^-1^. The light condition during shoot regeneration was a 16-h photoperiod. All experiments had three replications, each with three dishes. The shoot regeneration percentage was evaluated according to the percentage of embryos with calli regenerating shoots. Regeneration percentages of green and albino shoots were, respectively, the percentages of embryos with at least one green shoot and those with only albino shoots. Effects of light conditions during callus induction on shoot regeneration and endogenous hormone contents in calli were examined in plants grown in 2009. Treatments of ABA and fluridone, and gene expression analysis were examined in plants grown in 2014.

### Quantification of endogenous hormones in calli

Extraction, purification, and quantification of indole acetic acid (IAA), isopentenyladenine (iP), *trans*-zeatin (tZ), Gibberellin A1 (GA1), GA4, ABA, jasmonic acid (JA), jasmonyl isoleucine (JA-Ile), and salicylic acid (SA) from calli were conducted as described by Tsukahara *et al*. [42]. Calli derived from immature embryos cultured for two weeks were used for quantification in four cultivars. Approximately 100 mg fresh weight (FW) of calli was used in each of three independent experiments. Endogenous hormone levels in calli cultured with fluridone were determined in KN5 and LN.

### RNA isolation and quantitative RT-PCR analysis

For quantitative RT-PCR experiments, total RNA was isolated from 300 mg of calli derived from immature embryos cultured for two weeks in a 16-h photoperiod and continuous darkness using a kit (FastRNA Pro Green; Qbiogene Inc.). First, strand cDNA was prepared using a reagent kit (ExScript RT; TaKaRa). Then, PCR was performed using a LightCycler 2.0 (Roche Life Science) with SYBER Premix Ex Taq (TaKaRa). The following gene-specific primers designated by Millar *et al*. [43] were used: *HvABA8’OH-1*, A5F 5'-AGCACGGACCGTCAAAGTC-3', A5R 5'-TGAGAATGCCTACGTAGTG-3'; *HvNCED1* N10F 5'-CCAGCACTAATCGATTCC-3', N10R 5'-GAGAGTGGTGATGAGTAA-3'; *HvActin* HvActF 5'-AGGTGCCCTGAGGTCCTCTT-3', HvActR 5'-GTAGGTCGTCTCGTGGATTCCA -3' The relative amount of each target transcript was determined by generating standard curves using a dilution series of amplified products of the target sequence. Quantification was done using three biological replications. All quantifications were normalized to the amplification of *HvActin*. The accession numbers of *HvABA8’OH-1*, *HvNCED1*, and *HvActin* are, respectively, DQ145932, DQ145930, and AY14545. All kits were used according to the manufacturers’ respective protocols.

### Statistical analysis

Differences in mean values were tested using Student’s *t*-test or Duncan’s multiple range test.

## Results

### 1. Light control of shoot regeneration in barley cultivars

Immature embryos were cultured in a 16-h photoperiod and continuous darkness during the callus induction. In KN5, shoot regeneration percentages were, respectively, 25.2±4.3% and 61.8±6.5% in a 16-h photoperiod and continuous darkness (Table 1). The shoot regeneration percentage in a 16-h photoperiod was significantly lower than those in continuous darkness. Efficient shoot regeneration in continuous darkness was also observed in K3. However, shoot regeneration percentages of GP were, respectively, 79.3±4.8% and 54.7±2.9% in a 16-h photoperiod and continuous darkness. Shoot regeneration was inhibited by the culture in continuous darkness. Also, LN showed lower shoot regeneration percentage in continuous darkness. These cultivars were classified into photo-inhibition type (KN5 and K3) and photo-induction type (GP and LN) according to their response to light. No significant difference was found in albino shoot production between light conditions among all cultivars.

**Table 1:** Percentage of shoot regeneration in barley cultivars.

			Shoot regeneration (%)		
Cultivar	Light condition	No. of embryos	Green	Albino	Total
KN5	16-h photo	99	24.0±4.1**	1.1±1.4	25.2±4.3**
	Dark	108	58.3±8.5	3.5±2.2	61.8±6.5
K3	16-h photo	107	17.0±3.0*	5.0±3.3	22.0±5.8*
	Dark	108	46.3±8.8	3.8±2.4	50.1±7.0
GP	16-h photo	92	79.3±4.8**	0±0	79.3±4.8**
	Dark	104	53.7±2.8	1.0±1.3	54.7±2.9
LN	16-h photo	100	93.6±4.7*	2.2±2.7	95.8±2.6*
	Dark	109	73.3±8.3	0.9±1.1	74.2±7.6

16-h photo, 16-h photoperiod; dark, continuous darkness.

KN5, K3, GP, and LN respectively denote Kanto Nijo-5, K-3, Golden Promise, and Lenins.

Shoot regeneration percentages are expressed as mean±SE (n = 3).

*, **: Significantly different between light conditions at *P*<0.05 and *P*<0.01, respectively.

### 2. Endogenous hormone contents in calli

**Fig 1:**
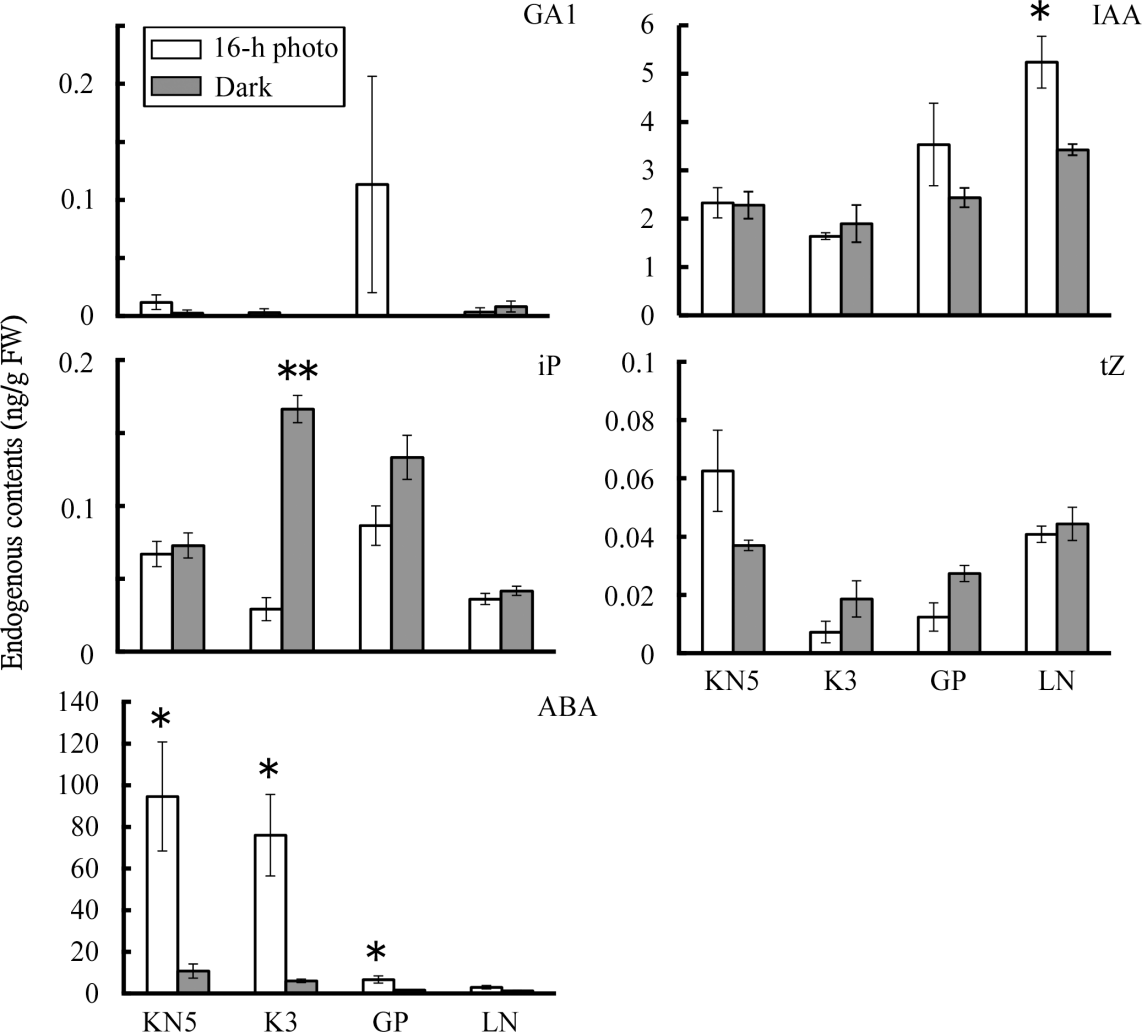
Endogenous hormone contents in calli. Endogenous hormone contents of GA1, IAA, iP, tZ, and ABA were determined in calli cultured under a 16-h photoperiod and continuous darkness during callus-induction. Error bars represent standard errors (n = 3). *, **: Significant difference between light conditions at *P*<0.05 and *P*<0.01, respectively.

Endogenous hormone contents were determined in calli cultured in a 16-h photoperiod and continuous darkness. Contents of GA1, tZ, and JAIle were low in calli, irrespective of light conditions (Fig 1 and S1 Fig). Although contents of iP were also low, K3 showed significantly higher iP contents in continuous darkness. GA4 was not detected in calli. Although contents of JA and SA differed among cultivars, no significant difference was found between light conditions. In KN5 and K3, endogenous IAA contents showed similar levels in both light conditions. However, LN showed significantly higher IAA contents in a 16-h photoperiod. Endogenous ABA contents in KN5 and K3 were 94.6±26.1 ng/g FW and 76.0±19.6 ng/g FW, respectively, in calli cultured in a 16-h photoperiod. However, calli cultured in continuous darkness showed low levels of ABA (6.1–10.8 ng/g FW). Endogenous ABA contents clearly differed between light conditions during callus induction. Although GP and LN showed higher endogenous ABA in a 16-h photoperiod, contents of endogenous ABA (3.0–6.8 ng/g FW) were lower levels similar to those of KN5 and K3 in continuous darkness. In this study, light conditions during callus induction strongly affected endogenous ABA contents in photo-inhibition type calli. Endogenous IAA contents were modified by light conditions only in the photo-induction type (GP and LN).

### 3. Effects of exogenous ABA and fluridone on tissue culture traits

**Fig 2:**
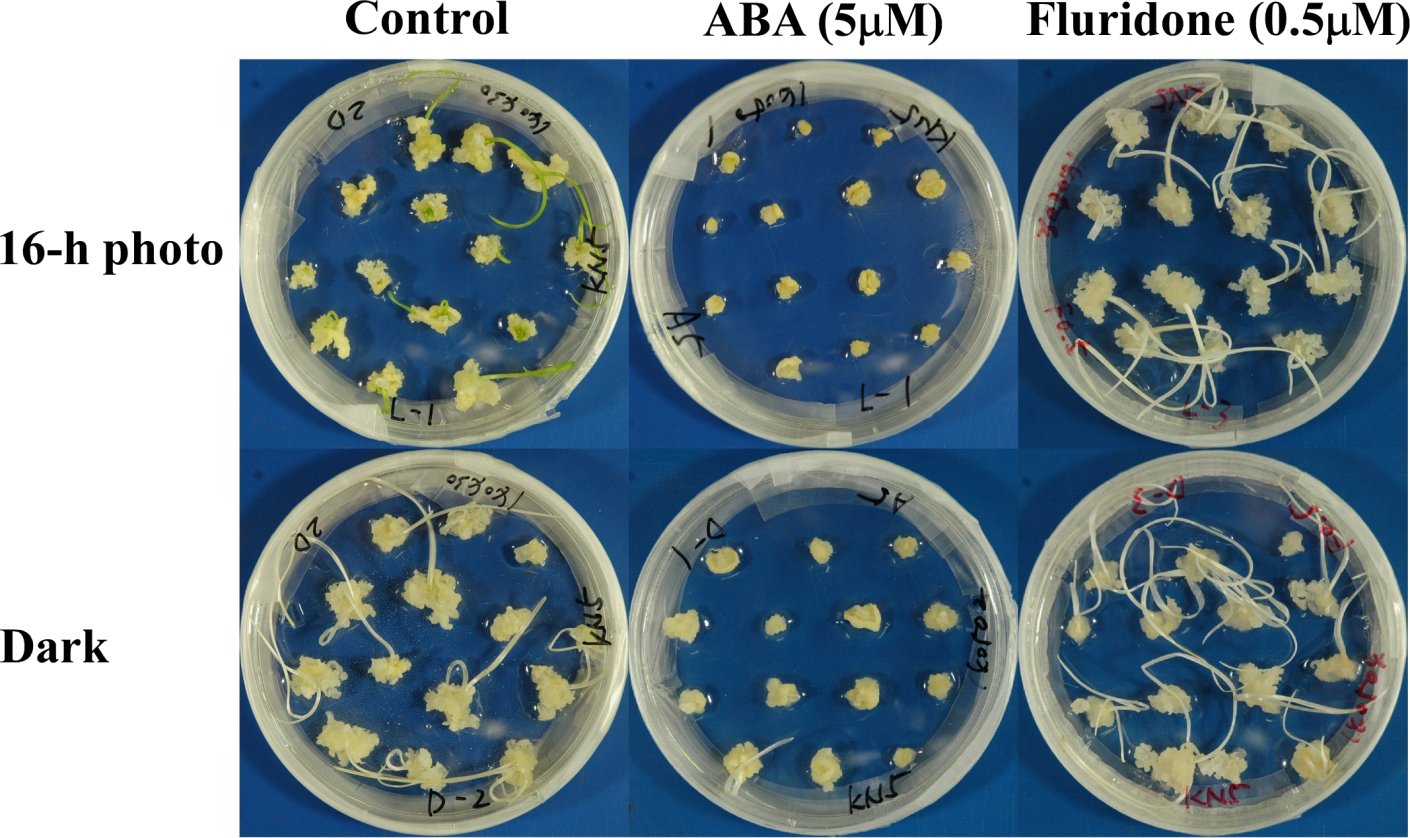
Effects of exogenous ABA and fluridone on callus growth. Immature embryos were cultured with ABA (5 μM) or fluridone (0.5 μM) in different light conditions (16-h photoperiod and continuous darkness) in Kanto Nijo-5.

**Fig 3:**
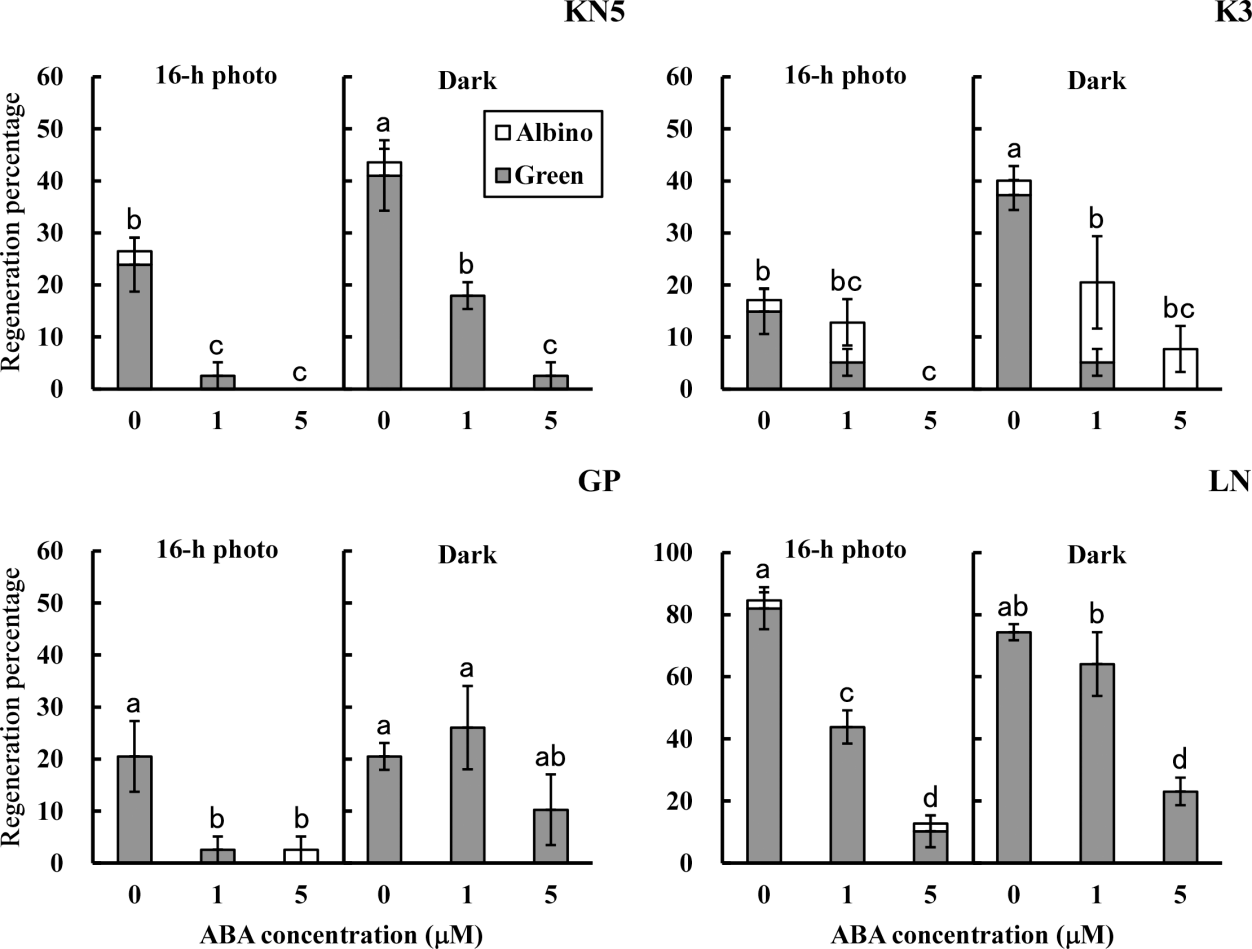
Effects of exogenous ABA on shoot regeneration. Percentages of green and albino shoot regeneration in calli cultured with or without ABA during callus-induction in different light conditions (16-h photoperiod and continuous darkness). Error bars represent standard errors (n = 3). Bars with different letters show a significant difference between total regeneration percentages (green and albino) by Duncan’s multiple range test (*P*<0.05).

Germination of zygotic embryos and callus growth were inhibited by exogenous ABA in both light conditions (Fig 2). In KN5 and GP, shoot regeneration was inhibited in cultures with ABA in a 16-h photoperiod (Fig 3). Total regeneration percentages (green and albino) were reduced depending on the concentration of ABA in continuous darkness. Reduction of total regeneration percentages depending on the concentration of ABA was also observed in cultures of K3 and LN in both light conditions. In all cultivars, shoot regeneration was inhibited by exogenous ABA. No significant difference was found in the inhibitory effects of exogenous ABA on shoot regeneration between light conditions.

**Fig 4:**
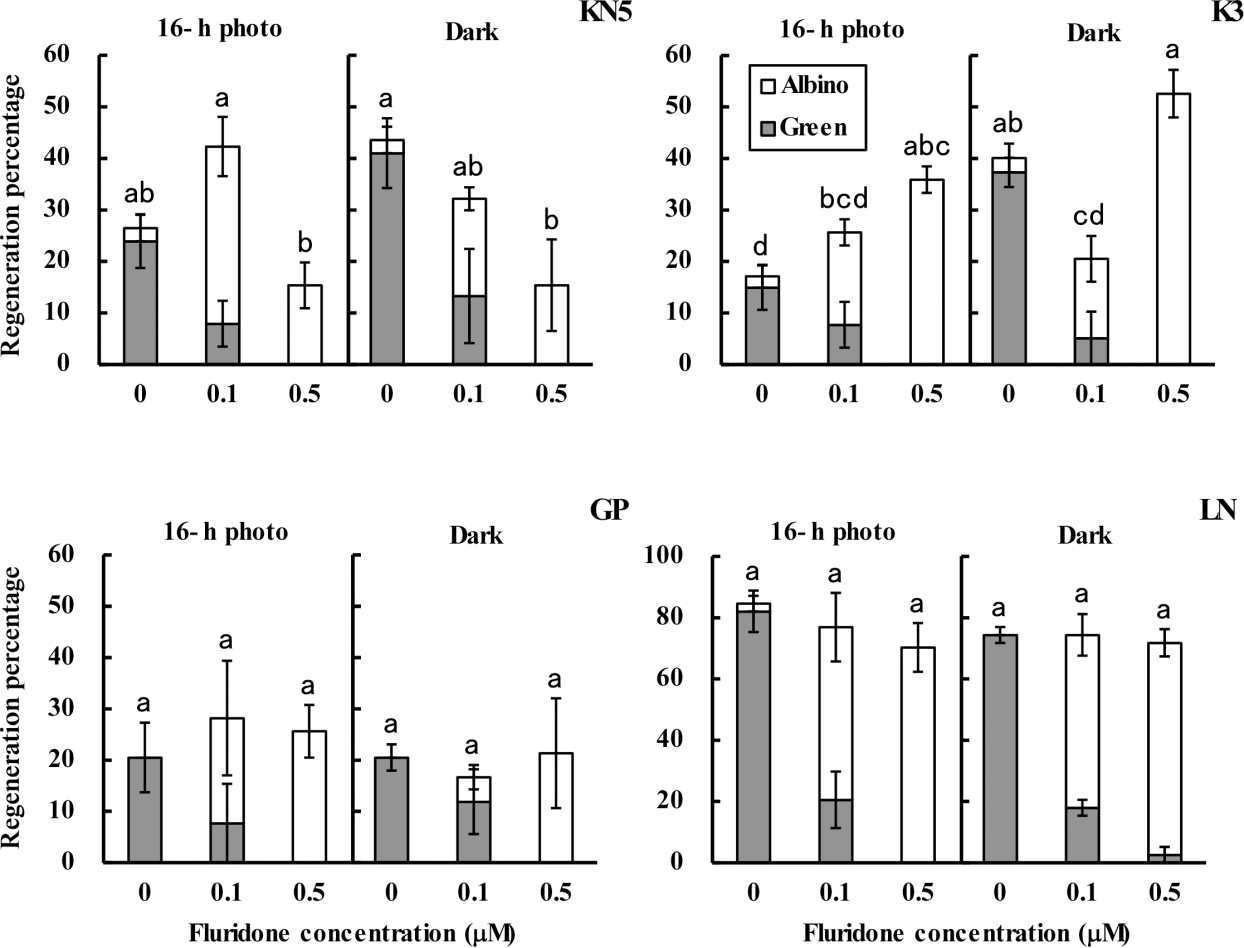
Effects of fluridone on shoot regeneration. Percentages of green and albino shoot regeneration in calli cultured with or without fluridone in different light conditions (16-h photoperiod and continuous darkness). Error bars represent standard errors (n = 3). Bars with different letters show a significant difference between total regeneration percentages (green and albino) by Duncan’s multiple range test (*P*<0.05).

An ABA biosynthesis inhibitor, fluridone, exhibited no marked effects on callus growth, whereas zygotic embryos germinated vigorously on the callus-induction medium with fluridone (Fig 2). Albino shoot production was enhanced strongly by the culture with fluridone in both light conditions. Total regeneration percentages of KN5 were increased at 0.1 μM of fluridone treatment and were decreased at 0.5 μM of treatment in a 16-h photoperiod (Fig 4). In continuous darkness, the total regeneration percentages decreased depending on the concentration of fluridone. In KN5, fluridone enhanced shoot regeneration at the lower concentration in a 16-h photoperiod and inhibited shoot regeneration in continuous darkness. In K3, total shoot regeneration percentages were increased depending on the concentration of fluridone in a 16-h photoperiod. In continuous darkness, although shoot regeneration percentages were decreased at 0.1 μM of fluridone treatment, a higher shoot regeneration percentage was observed at 0.5 μM of fluridone treatment. Fluridone enhanced shoot regenerations of KN5 and K3 in a 16-h photoperiod. However, although albino shoot regenerations were also enhanced by fluridone in GP and LN, no significant effect was observed in the total shoot regeneration percentage. The response to fluridone differed between the photo-inhibition type and the photo-induction type.

### 4. Endogenous hormone contents in calli cultured with fluridone

**Fig 5:**
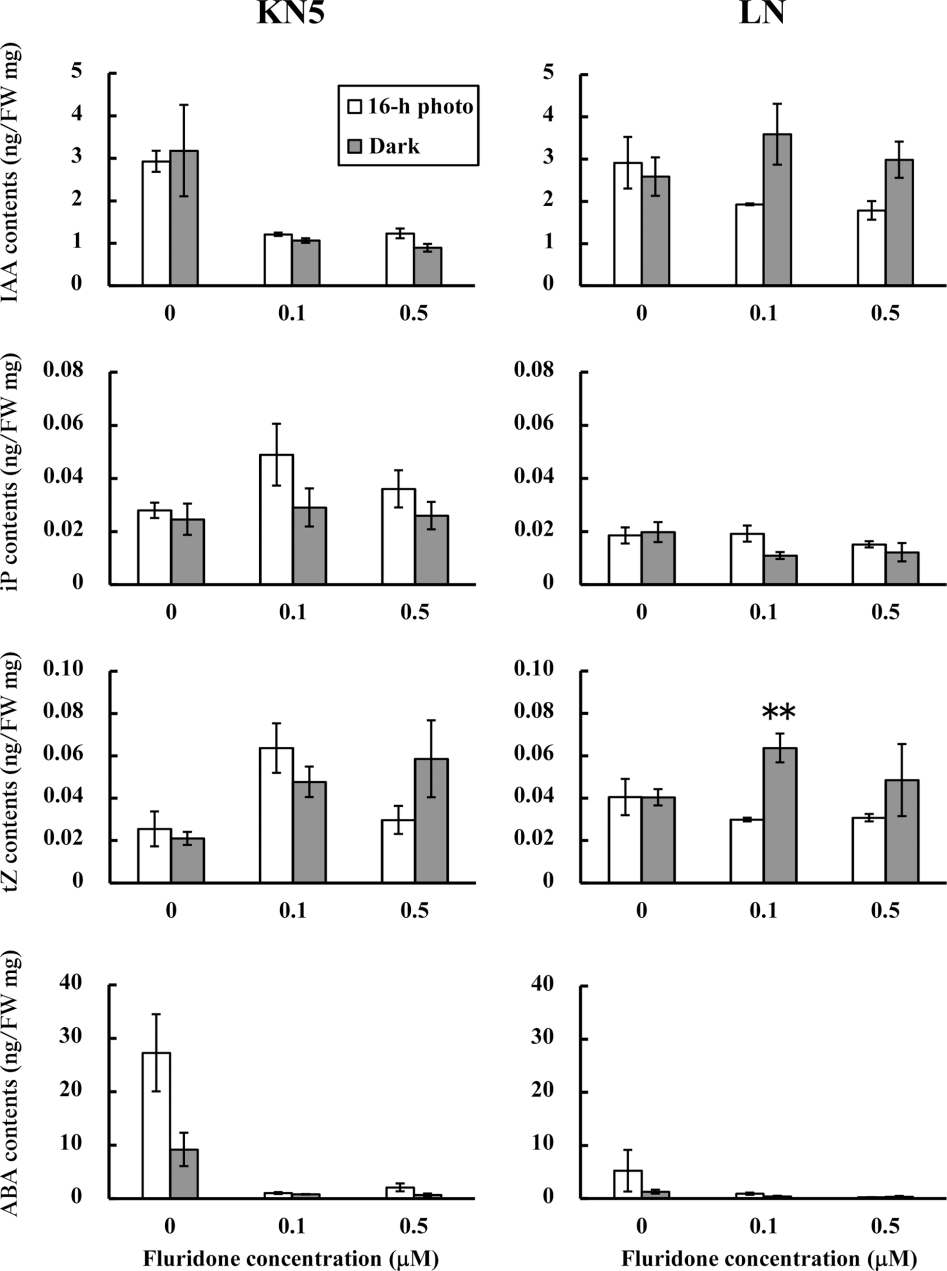
Effects of light conditions on endogenous hormone contents in calli under varied fluridone concentrations. Endogenous hormone contents of IAA, iP, tZ, and ABA were determined in calli cultured with or without fluridone in different light conditions (16-h photoperiod and continuous darkness). Error bars represent standard errors (n = 3). **: Significant difference between light conditions at *P*<0.01.

Endogenous hormone contents were examined in calli cultured with fluridone in KN5 and LN. Contents of tZ and iP were low in calli. The effects of fluridone were unclear (Fig 5). Fluridone had no effect on the endogenous level of SA. Moreover, GA1, JA and JAIle showed low contents in calli (data not shown). Endogenous contents of ABA were reduced strongly in cultures with fluridone in both light conditions. Furthermore, fluridone treatments decreased endogenous IAA contents in KN5, irrespective of light conditions. Fluridone had no significant effect on endogenous IAA content of LN in continuous darkness. However, endogenous IAA contents of LN were lower in the cultures with fluridone in a 16-h photoperiod (0.05<*P*<0.10). Cultures with fluridone during the callus-induction affected contents of ABA and IAA in calli.

### 5. Expressions of ABA catabolism and biosynthesis genes in calli

**Fig 6:**
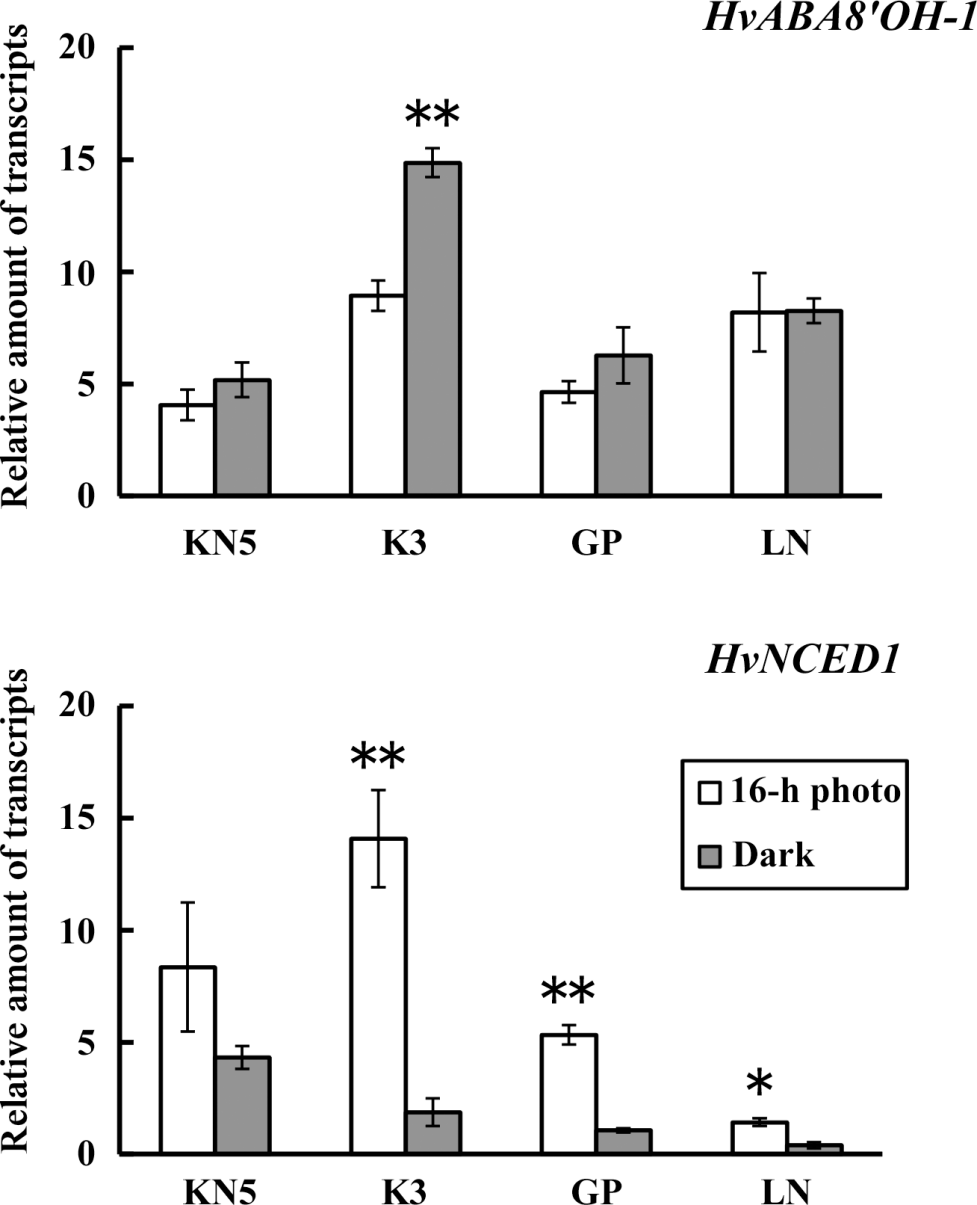
Relative amounts of transcripts of *HvABA8’OH-1* and *HvNCED1* in calli. Transcripts were determined in calli cultured under different light conditions (16-h photoperiod and continuous darkness). Error bars represent standard errors (n = 3). *, **: Significant difference between light conditions at *P*<0.05 and *P*<0.01, respectively.

Expressions of *HvABA8’OH-1* and *HvNCED1*, which were respectively associated with ABA catabolism and biosynthesis, were examined in calli. Expressions of *HvABA8’OH-1* showed similar levels in a 16-h photoperiod and continuous darkness in KN5, GP, and LN. No significant effect of light conditions was detected in the regulation of *HvABA8’OH-1* expression in these cultivars (Fig 6). However, *HvABA8’OH-1* was highly expressed in continuous darkness in K3. *HvABA8’OH-1* expression represented light dependency independent of photo-regulation type for shoot regeneration.

Expressions of *HvNCED1* were significantly higher in a 16-h photoperiod than in continuous darkness in K3, GP, and LN (Fig 6). Expression levels differed among cultivars in a 16-h photoperiod. The photo-inhibition type showed higher expressions than the photo-induction type. Expression of *HvNCED1* in calli was regulated by the light condition during callus induction.

## Discussion

Shoot regeneration is regulated by light conditions that prevail during callus induction in immature barley embryo culture. The responsiveness to light differs among cultivars [40]. In a 16-h photoperiod, shoot regeneration of KN5 and K3 (photo-inhibition type) was inhibited. In contrast, shoot regenerations of GP and LN (photo-induction type) were enhanced in a 16-h photoperiod. These results support those of an earlier study [40]. For investigation of the regulatory mechanisms of light in immature barley embryo culture, endogenous hormone contents were examined in calli cultured under a 16-h photoperiod and continuous darkness. Higher accumulations of ABA were observed in a 16-h photoperiod in photo-inhibition type, although the levels of ABA were lower in continuous darkness. Exogenous ABA supplemented with the callus-induction medium inhibited callus growth and shoot regeneration. Furthermore, ABA biosynthesis inhibitor, fluridone, decreased endogenous ABA in calli. Photo-inhibition of shoot regeneration was reduced in KN5 and K3. These results indicate that the photo-inhibition of shoot regeneration is associated with the fluctuation of endogenous ABA contents in KN5 and K3.

The effects of ABA on tissue culture traits in several species have been investigated. Rajasekaran *et al*. [44] reported that exogenous application of ABA enhanced somatic embryogenesis and that it reduced the formation of non-embryogenic callus in Napier grass. Positive effects of ABA on shoot regeneration were also observed in calli derived from immature embryos of wheat [45, 46], embryos and anthers of rice [6, 47, 48], cotyledons of rape [4, 5], hypocotyls of carrot [2], and immature embryos of coconut [49]. However, ABA acts as a negative factor affecting shoot regeneration in calli derived from immature embryos of corn [50], leaf explants of alfalfa [51], immature seeds of Hevea [52], immature seeds of sunflower [53], and nodal segments of peach [54]. In the present study, ABA inhibits shoot regeneration in calli derived from immature barley embryos, whereas ABA promotes plant regeneration in barley anther culture [55]. Consequently, the effects of ABA on tissue culture traits differ depending on the species and explant source. In rice and cactus, callus growth was enhanced by low concentration of ABA; it was inhibited by high concentration [7, 56]. Furthermore, somatic embryo formation and plant regeneration were enhanced by low concentration of ABA and were inhibited by high concentrations in sweet orange and melon [3, 57]. ABA is necessary for shoot regeneration at a low concentration. A higher concentration of ABA might act as an inhibitor on tissue culture traits. However, Qureshi *et al*. [58] reported that ABA inhibited shoot regeneration in calli derived from wheat embryos in an early stage (10–14 days after pollination; DAP), whereas shoot regeneration was enhanced in calli derived from late stage embryos (21–25 DAP) through the inhibition of precocious germination. The developmental stage of seed affects sensitivity to ABA in wheat [59]. Recently, *receptor like protein kinase 1* (*RPK1*) was identified as a QTL for the regulation of plant regeneration in Arabidopsis; more importantly, *RPK1* is involved in the ABA signal transduction pathway [23]. Zhang *et al*. [24] reported that expressions of several miRNA are decreased in the embryogenic calli of *Larix leptolepis* and reported that the targets of these miRNA are positive regulators of ABA responses. These results indicate that ABA signals are involved in regulating shoot regeneration. Sensitivity to ABA might be associated with different responses of tissue culture traits.

In expression analysis, the expression of *HvNCED1* was increased in a 16-h photoperiod. The *NCED* encodes 9-*cis*-epoxycarotenoid deoxygenase, which is a major enzyme for ABA biosynthesis, and acts at a key regulatory step in ABA biosynthesis. Thompson *et al*. [60] reported that the expression of *NCED* was increased in the light period and decreased in the dark period of a 12-h photoperiod in tomato. Moreover lower expressions were maintained in continuous darkness followed by a 12-h photoperiod. In tartary buckwheat, sprout of Hokkai T10 showed higher expressions of *NCED* in a 16-h photoperiod and lower expressions in continuous darkness [61]. *HvNCED1* is also positively regulated by light in calli derived from immature barley embryos. In the photo-inhibition type, the increase of *HvNCED1* expression activates ABA biosynthesis and induces high accumulation of endogenous ABA. Eventually, higher accumulation of ABA inhibits shoot regeneration. Rikiishi *et al*. [40] reported that blue light signals act in photo-inhibition of shoot regeneration in immature barley embryo culture. Expression of *HvNCED1* might be regulated by blue light signals.

In this study, immature embryos were derived from plants grown in different years. Effects of light conditions during callus-induction on shoot regeneration (Table 1) and endogenous hormone contents in calli (Fig 1 and S1 Fig) were examined in plants grown in 2009, whereas effects of exogenous ABA and fluridone (Figs 2–5) and the analysis of gene expression (Fig 6) were examined in plants grown in 2014. Although different levels of shoot regeneration percentage were observed in our experiments, light conditions affected the shoot regeneration in KN5 and K3. KN5 showed different levels of endogenous ABA contents in calli cultured in a 16-h photoperiod (94.6±26.1 ng/g FW in Fig 1; 27.3±7.2 ng/g FW in Fig 5). However, endogenous ABA contents were higher in a 16-h photoperiod than in continuous darkness, as shown in Fig 2 (*P*<0.05) and Fig 5 (0.05<*P*<0.10). These results indicate that photo-inhibition type shows marked response to light conditions in distinct experiments. In this study, plants were grown in a field, not in a growth chamber. The quality of immature embryos is affected strongly by the vegetative conditions of the mother plants [33]. Different levels of shoot regeneration percentage and endogenous hormone content might be derived from the growth conditions of plants. However, effects of light conditions were not detected in photo-induction type in Fig 3 and Fig 4. Sensitivity to light conditions might be different between photo-inhibition and photo-induction types. Lower sensitivity induces unstable conditions of the photo-induction type.

In the photo-induction type, the reduction of endogenous ABA contents by fluridone showed no effects on the shoot regeneration, although exogenous ABA inhibited plant regeneration similarly to the photo-inhibition type. Because GP and LN had innately lower concentrations of ABA in both light conditions, photo-induction of shoot regeneration was independent of the endogenous ABA. Auxin-related and cytokinin-related processes are important for shoot regeneration and are highly conserved in plants [23]. In this study, endogenous IAA contents were reduced in continuous darkness in LN. In photo-induction type, shoot regeneration might be regulated by light through the auxin function.

Endogenous ABA content was reduced by the cultures with fluridone, independent of the light conditions. Fluridone reduces ABA contents and affects chloroplast development because fluridone inhibits the desaturation of phytoene at the early step of carotenoid biosynthesis [62–64]. Hoekstra *et al*. [65] reported that the production of albino shoot regeneration was enhanced strongly by fluridone in barley anther culture. Albino shoot regeneration was increased in calli cultured with fluridone in this study. However, shoot regeneration percentages were reduced in cultures with higher concentrations of fluridone. Nevertheless, higher concentrations had no greater effect on the reduction of endogenous ABA. Fluridone has inhibitory effects on shoot regeneration independent of ABA biosynthesis. Other inhibitors are expected to be necessary for the investigation of ABA effects on shoot regeneration. Han *et al*. [66] developed an inhibitor of NCED. Our results show that photo-inhibition of shoot regeneration is regulated by NCED expression. Endogenous ABA might be reduced by an inhibitor of NCED in avoiding albino shoot production. The effects of ABA on shoot regeneration can be evaluated precisely in immature barley embryo culture.

Shoot regeneration from de-differentiated tissues is an important step in the production of transgenic plants and for demonstrating totipotency. Rikiishi *et al*. [40] reported that the genes responsive to auxin and cytokinin showed different expression ratios depending on the light conditions in calli of KN5 and K3. This result indicates that auxin and cytokinin have an important role in the regulation of shoot regeneration in immature barley embryo culture. Results of this study show that ABA acts as an inhibitor of shoot regeneration in calli derived from immature embryos. This study also elucidates the role of ABA for the photo-regulation of shoot regeneration. Shoot regeneration is controlled by a regulatory network involving crosstalk among auxin, cytokinin, and ABA. Auxin and cytokinin have been investigated and understood in the function of regulating tissue culture traits. However, the physiological functions of ABA on tissue culture traits remain unclear. Endogenous contents of ABA and shoot regeneration capacity can be modified by simple light signals, suggesting that immature barley embryo culture is a useful tool for investigating the relation between ABA and tissue culture traits.

## Supporting Information

S1 Fig Endogenous hormone contents in calli. Endogenous hormone contents of JA, JAIle and SA were determined in calli cultured under a 16-h photoperiod and continuous darkness during callus-induction. Error bars represent standard errors (n = 3). *: Significantly different between light conditions at *P*<0.05.
(TIF)
