# Supplementary figures and images for: Light Inhibition of Shoot Regeneration Is Regulated by Endogenous Abscisic Acid Level in Calli Derived from Immature Barley Embryos
Source: PLoS One. 2015 Dec 15;10(12):e0145242. doi: 10.1371/journal.pone.0145242 (PMC4682856; doi:10.1371/journal.pone.0145242)

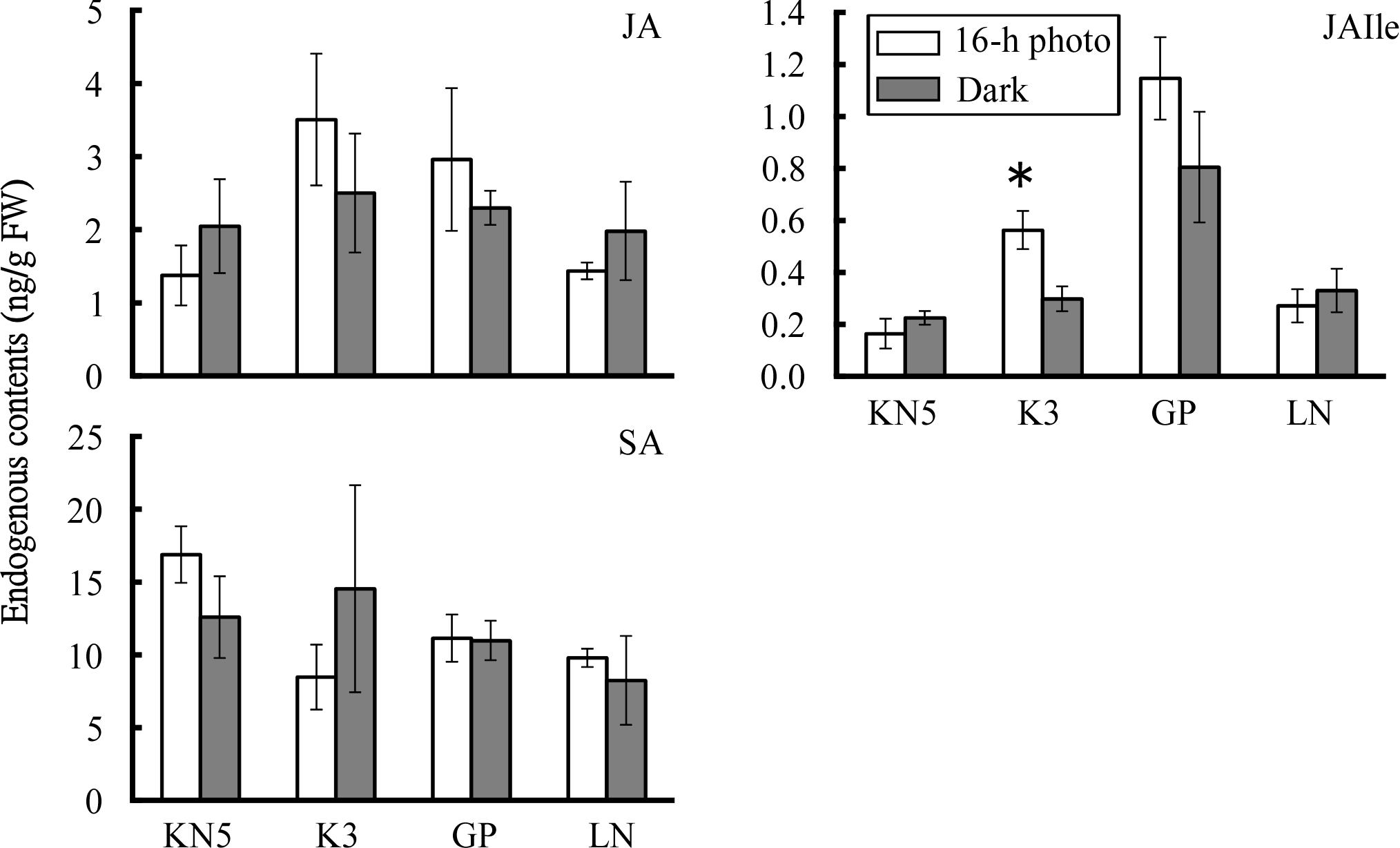

Supplement: S1 Fig — Endogenous hormone contents of JA, JAIle and SA were determined in calli cultured under a 16-h photoperiod and continuous darkness during callus-induction. Error bars represent standard errors (n = 3). *: Significantly different between light conditions at P<0.05. (TIF) [file pone.0145242.s001.tif]
